# Bioprinted Scaffolds for Biomimetic Applications: A State-of-the-Art Technology

**DOI:** 10.3390/biomimetics10090595

**Published:** 2025-09-05

**Authors:** Ille C. Gebeshuber, Sayak Khawas, Rishi Sharma, Neelima Sharma

**Affiliations:** 1 Institute of Applied Physics, Vienna University of Technology, A-1040 Vienna, Austria; gebeshuber@iap.tuwien.ac.at; 2 Department of Pharmaceutical Sciences and Technology, Birla Institute of Technology, Mesra, Ranchi 835215, India; sayakkhawas96@gmail.com; 3 Department of Physics, Birla Institute of Technology, Mesra, Ranchi 835215, India; rishisharmabit@hotmail.com

**Keywords:** 3D bioprinting, biomimetic scaffolds, bioinks, tissue engineering, regenerative medicine

## Abstract

This review emphasizes the latest developments in bioprinted scaffolds in tissue engineering, with a focus on their biomimetic applications. The accelerated pace of development of 3D bioprinting technologies has transformed the ability to fabricate scaffolds with the potential to replicate the structure and function of native tissues. Bioprinting methods such as inkjet, extrusion-based, laser-assisted, and digital light processing (DLP) approaches have the potential to fabricate complex, multi-material structures with high precision in geometry, material composition, and cellular microenvironments. Incorporating biomimetic design principles to replicate the mechanical and biological behaviors of native tissues has been of major research interest. Scaffold geometries that support cell adhesion, growth, and differentiation essential for tissue regeneration are mainly of particular interest. The review also deals with the development of bioink, with an emphasis on the utilization of natural, synthetic, and composite materials for enhanced scaffold stability, printability, and biocompatibility. Rheological characteristics, cell viability, and the utilization of stimuli-responsive bioinks are also discussed in detail. Their utilization in bone, cartilage, skin, neural, and cardiovascular tissue engineering demonstrates the versatility of bioprinted scaffolds. Despite the significant advancements, there are still challenges that include achieving efficient vascularization, long-term integration with host tissues, and scalability. The review concludes by underlining future trends such as 4D bioprinting, artificial intelligence-augmented scaffold design, and the regulatory and ethical implications involved in clinical translation. By considering these challenges in detail, this review provides insight into the future of bioprinted scaffolds in regenerative medicine.

## 1. Introduction

The field of tissue engineering is being transformed by the integration of new approaches to scaffold fabrication. Thus, 3D bioprinting has emerged as a transformative technology in tissue engineering and plays a central role in advancing regenerative medicine. This technology enables the creation of biomimetic scaffolds in precise detail to mimic the native tissue extracellular matrix (ECM) and provide a better form of tissue repair and regeneration than traditional approaches. The ability to create scaffolds that can mimic the complex topology and function of native tissues is at the heart of improving the efficacy of regenerative therapies and reducing some of the shortcomings of current therapies for tissue damage and organ failure [1,2].

During the past few years, significant progress has been made in the development of bioprinted scaffolds. Scaffolds are made up of biomaterials, bioactive molecules, and living cells that interact to form functional constructs and could induce cellular growth and differentiation. Progress in bioink formulation, scaffold topology, and print technology has allowed for the development of more sophisticated, patient-specific constructs. With the application of 3D bioprinting, it has been feasible to control the distribution of cells, materials, and growth factors precisely to enable tissue regeneration, and the scaffolds are valuable tools in the construction of personalized medication and surgery [3,4].

One of the biggest challenges in tissue engineering has been the development of scaffolds that can support tissues without compromising their structural integrity and functionality over time. Recent studies have highlighted the importance of biomimetic scaffolds that are designed to replicate the mechanical and biochemical properties of native tissues. These scaffolds provide not only physical support but also induce cellular activities such as cell migration, differentiation, and angiogenesis, which are essential for effective tissue repair and regeneration [5]. Incorporation of bioactive molecules such as growth factors into the scaffolds further enhances their ability to support tissue regeneration by inducing cellular responses [1].

Recent advances in 3D bioprinting have greatly improved the precision and complexity of scaffold printing, enabling the fabrication of structures composed of more than a single cell type, with mechanical properties and functional gradients. These advances have proven to be promising in skin, bone, cartilage, and vasculature repair. Despite all of this, critical challenges such as adequate vascularization, long-term viability, and integration with host tissue remain at the heart of the clinical efficacy of such bioprinted structures. Despite these limitations, the potential of 3D bioprinted biomimetic scaffolds to advance tissue engineering remains considerable, and ongoing research continues to address existing challenges to realize their full therapeutic potential [3,5].

Recent research has largely enhanced the design of bioprinted scaffolds, such as bioink formulation and printing techniques. Zoghi, in 2024, presented a comprehensive review on the history of tissue engineering, including the addition of bioprinting techniques, scaffold production, and bioinks for tissue repair [1]. Similarly, Mirsky et al. (2024) presented a comprehensive review of 3D bioprinting, with a focus on its use in regenerative medicine and tissue engineering [2]. Liu et al. (2022) also presented an overview of the targeted tissue regeneration applications of 3D bioprinted scaffolds and materials used for their production [3]. Górnicki et al. (2024) demonstrated new approaches in biomimetic scaffold design, which may improve tissue culture techniques and scaffold integration [4]. The current state of 3D bioprinting in skin tissue engineering presents key insights into its application in clinical use [5].

This review highlights the most recent advances in the design, fabrication, and use of bioprinted scaffolds in tissue engineering. Through the focus on the most recent advances in bioinks, scaffold architectures, and printing methods, we try to provide a clear view of the current challenges and opportunities in the field, and the future of 3D bioprinting for biomimetic purposes.

While several reviews have addressed aspects of 3D bioprinting, most have either focused broadly on the technology or targeted specific tissues. What remains less explored is a comprehensive perspective on the incorporation of biomimetic principles into scaffold design, specifically how the geometry, material choice, and bioink formulation contribute to replicating the structural and functional features of native tissues. This review aims to fill that gap by critically discussing the most recent advances in bioprinted scaffolds, their biomimetic applications across multiple tissues, and emerging directions such as 4D bioprinting and AI-assisted scaffold design. Thus, this review provides a timely and significant contribution by positioning biomimetic strategies as the central framework for scaffold design in regenerative medicine.

## 2. Fundamentals of 3D Bioprinting

### 2.1. Historical Evolution of Bioprinting Technologies

The roots of printing can be traced back to woodblock methods used in China before 220 A.D [6], culminating in the revolutionary invention of the printing press in 15th century Europe [7]. These technological leaps transformed the dissemination of information across disciplines. In the modern era, printing has evolved into three-dimensional (3D) fabrication through additive manufacturing (AM), which constructs objects layer by layer. Initially prominent in aerospace and architectural fields, 3D printing has expanded into personalized consumer goods and biomedical domains.

The conceptual framework of 3D printing was introduced by David E. H. Jones in 1974, and was technically implemented by Hideo Kodama in 1981 using photo-curable polymers. The field advanced with Charles W. Hull’s invention of stereolithography in 1986, which enabled precise layer-by-layer photopolymerization. Further developments in printing biological materials without toxic solvents led to the emergence of bioprinting [8].

Bioprinting is a specialized branch of AM that involves the layer-wise deposition of viable cells, biomaterials, and biological molecules to fabricate tissue-like constructs. Unlike traditional post-fabrication cell seeding, bioprinting integrates cells during scaffold fabrication, enabling homogeneous cell distribution. This method has shown improved integration with host tissues, uniform tissue development, and reduced immunological rejection [9].

A key challenge in scaffold design is maintaining a balance between mechanical integrity and biological compatibility. Bioprinted constructs must support cell ingrowth while avoiding cytotoxic effects from materials or stress-induced apoptosis during extrusion. Compared to conventional static and dynamic seeding methods, which may compromise the cell morphology, bioprinting offers superior control over cell placement and structure fidelity [10].

Vascularization remains a critical hurdle in tissue engineering. Bioprinting facilitates the fabrication of pre-vascularized scaffolds by enabling the inclusion of microvascular-like structures and the precise positioning of endothelial cells. Such constructs ensure adequate oxygen and nutrient supply, removal of metabolic waste, and prevention of necrosis, all of which are essential for tissue survival and remodeling [11].

Clinically, bioprinting holds promise for producing patient-specific regenerative scaffolds. Imaging techniques such as CT, MRI, or ultrasound can be used to generate a 3D model of the defect, which is then digitally customized using computer-aided design (CAD). Based on the anatomical and physiological requirements of the defect, appropriate biomaterials, cell types, and bioactive molecules are selected to formulate a bioink. This bioink is then used to fabricate a tailored construct, which may undergo maturation in vitro or be directly implanted [12].

### 2.2. Key Bioprinting Modalities

**Figure 1:**
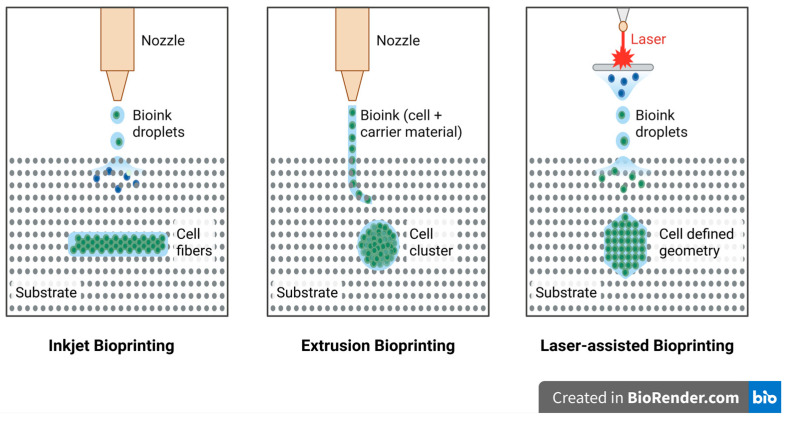
Bioprinting Techniques.

Currently, 3D bioprinting is seen as a promising technique for the direct deposition of living cells into complex three-dimensional structures through a top-down fabrication approach. Among the available techniques, inkjet bioprinting, laser-assisted bioprinting, and extrusion bioprinting are the three prominent modalities, as illustrated in Figure 1. However, none of the bioprinting techniques are yet ready to print synthetic tissues and organs of any size and structural complexity. Therefore, all these techniques need to be investigated thoroughly based on key performance parameters, such as the printing resolution, cell viability, and bioink material compatibility, to determine whether they would be suitable for application in particular tissue engineering applications [8]. Table 1 summarizes the different bioprinting techniques based on various properties such as material type, viscosity, printing cost, resolution, and cell viability.

#### 2.2.1. Inkjet Bioprinting

Inkjet bioprinting, one of the earliest approaches used for the deposition of live cells, was initially explored by modifying commercially available inkjet printers [7]. Early challenges in this technique included significant cell death due to rapid drying once deposited onto the substrate. This was later addressed by encapsulating the cells within highly hydrated polymers, giving rise to the development of cell-laden hydrogels that maintained cellular viability [13]. Inkjet bioprinting operates by ejecting droplets of bioink, comprising cells and biomaterials, through either thermal or piezoelectric mechanisms to precisely pattern them into desired geometries [14,15].

In thermal-based systems, a heating element rapidly creates a vapor bubble that builds pressure and forces the droplet out of the nozzle, reaching localized temperatures as high as 100 °C to 300 °C [16]. Despite initial concerns regarding cellular damage due to these temperatures, subsequent research revealed that the exposure was both brief and localized, thereby preserving cell viability. In contrast, piezoelectric-based systems use acoustic waves to eject droplets, avoiding thermal exposure but limiting the use of viscous bioinks due to the dampening effect of their viscosity on the pressure waves. This necessitates the use of low-concentration solutions, which can be a limitation in achieving mechanically stable 3D structures [17,18].

Although inkjet printing faces issues such as unreliable droplet directionality, limited vertical build-up, and low-viscosity requirements, it remains advantageous for its high resolution (up to 50 µm), speed, cost-effectiveness, and ability to achieve high cell viability with minimal shear-induced damage. Notable studies have demonstrated the utility of inkjet bioprinting in various tissue engineering applications. Cui et al. successfully fabricated bone-like scaffolds using PEGDMA and bioceramic nanoparticles such as hydroxyapatite and bioglass, which not only improved the compressive strength but also promoted osteogenic differentiation of human mesenchymal stem cells, with viability rates reaching up to 86% [10,18,19].

In the neural domain, Tse et al. have used piezoelectric bioprinting to deposit porcine Schwann cells and NG108-15 neuronal cells, reporting immediate post-printing viability rates of 86% and 90%, respectively, along with neurite extension over seven days [20]. Cardiac tissues engineered using alginate hydrogels and feline cardiomyocytes displayed contractility upon mild electrical stimulation, highlighting functional integration. Furthermore, full-thickness skin models with freckle-like pigmentation were produced in a study through the layered deposition of fibroblasts, keratinocytes, and melanocytes, resulting in successful dermal–epidermal stratification [5,15].

Despite its limitations, the high resolution, cell viability, and multi-cell patterning capabilities of inkjet bioprinting underscore its relevance in the field. However, its full potential may best be realized in conjunction with other complementary bioprinting techniques [21].

#### 2.2.2. Extrusion-Based Bioprinting

Extrusion bioprinting is a widely adopted pressure-driven technique that allows for the deposition of bioink in a continuous flow, creating 3D structures. Unlike inkjet bioprinting, which deposits liquid droplets, extrusion bioprinting uses pneumatic or mechanical pressure, such as piston- or screw-based systems, to extrude bioink through a nozzle [15]. The extruded material solidifies on the substrate, forming layers until the final 3D construct is completed. This method is advantageous because it can print materials with high viscosity and high cell densities, which is critical for applications requiring complex tissue structures. The process is enhanced by mechanical dispensing systems, which provide better control over the material flow, offering greater precision than pneumatic systems due to less delay in the compressed gas volume. Factors such as the viscosity, extrusion speed, cross-linking ability, and material properties of the bioink are important in achieving successful prints [22,23].

Extrusion-based printing allows high-density cell deposition, which is beneficial in creating structures with strong mechanical support or suitable environments for cell function. High-viscosity bioinks offer structural integrity, while low-viscosity inks create environments more conducive to cell viability. This flexibility is one of the key advantages of extrusion bioprinting. However, extrusion printing can negatively impact cell viability due to the shear stress during the extrusion process, leading to cell apoptosis and a reduction in the overall number of viable cells. The viability typically ranges from 40 to 86%, and it decreases with increasing extrusion pressure and smaller nozzle gauges. While it is less precise than inkjet-based bioprinting, extrusion bioprinting can still achieve good cell viability with appropriate process adjustments, such as optimizing the extrusion speed and bioink properties [7,24].

Extrusion bioprinting is a promising method for creating biomimetic structures and tissue engineering applications. Despite its lower resolution compared to inkjet bioprinting, it offers advantages in cell density and material versatility. Research on self-healing hydrogels and interpenetrating polymer networks is helping improve the print fidelity and cell viability, making this technique more applicable to a range of biological and medical fields [10].

#### 2.2.3. Laser-Assisted Bioprinting

Laser-assisted bioprinting (LAB) is a variation of laser-induced forward transfer and direct-write approaches that have a “ribbon” geometry for the print [25,26]. The geometry is a layer of energy-absorbing material (e.g., gold or titanium) over a bioink layer, which can have cells or hydrogel. Pulsed laser light stimulates the energy-absorbing layer when printing, which vaporizes and forms a high-pressure bubble that propels the bioink onto a receiving substrate as droplets. The print quality of the fabricated construct depends on multiple factors, including the wavelength, intensity, and pulse duration of the laser, as well as the surface tension, viscosity, and thickness of the bioink. Additionally, the substrate wettability and air gap between the substrate and the ribbon also play a critical role in determining printing precision [27,28].

Compared to conventional bioprinting techniques, LAB is a nozzle-free and contactless method, excluding mechanical stress on cells during printing and preserving their viability. This feature enables LAB to print a wide range of biological materials (for example, those with high viscosities or high-density cells), without impairing cell function. LAB is resistant to nozzle clogging, a limitation that other methods face. Setting up the ribbon setup for different cell types or materials is time-consuming, however, especially when co-depositing multiple cell types. Moreover, the effect of laser exposure on cells is not yet clear, and laser system operation is more complex than that of nozzle-based methods, making it difficult to deposit cells with accuracy [29,30,31]. LAB has a resolution range of 10–50 µm, and studies have demonstrated high cell viability (>95%) and accuracy, with the ability to deposit single cells per droplet. For instance, mesenchymal stem cells (MSCs) printed through LAB showed no significant change in gene expression, and their proliferation rates were like non-printed cells. LAB has been applied in tissue regeneration (for instance, skin and bone), where it has demonstrated potential in in vivo studies. Although LAB has its benefits, it has its drawbacks, such as high cost, low stability, and scalability limitations. However, when combined with other biofabrication techniques, LAB has vast potential for future applications in regenerative medicine [32,33].

#### 2.2.4. Digital Light Processing (DLP) Bioprinting

Alongside inkjet, extrusion, and laser-assisted methods, digital light processing (DLP) bioprinting has become an important approach for the fabrication of biomimetic scaffolds. DLP is a vat polymerization technique that relies on projecting a two-dimensional light pattern onto a photocurable bioink. A digital micromirror device or liquid crystal display directs ultraviolet or visible light to induce polymerization across the entire layer simultaneously, which is repeated in a layer-by-layer manner to generate the three-dimensional construct. Unlike stereolithography, which scans point by point, DLP photopolymerizes an entire slice at once, resulting in much faster processing and higher resolution [34].

The advantage of DLP lies in its ability to fabricate highly precise structures with excellent surface finish and geometric fidelity. Micron-level resolution can be achieved, which is especially useful for printing complex scaffold designs such as gyroid networks, lattice architectures, and perfusable channels. These fine features play a critical role in guiding cellular organization, nutrient diffusion, and vascularization within engineered tissues. The capacity to reproduce small and intricate designs makes DLP attractive for bone, cartilage, and vascular tissue engineering applications, where the geometry of the microenvironment strongly influences functional outcomes [35].

In terms of material compatibility, DLP is limited to photo-crosslinkable bioinks. Commonly used materials include gelatin–methacryloyl, polyethylene glycol diacrylate, and other chemically modified hydrogels. These inks can be optimized to provide the appropriate balance of mechanical stability, biological activity, and printability. However, as the method relies on light exposure, there is a need to carefully adjust the intensity and duration of illumination, as well as the photoinitiator concentration, in order to minimize cytotoxic effects. Despite this limitation, DLP-printed hydrogels often achieve high cell viability and support long-term proliferation [35].

Recent work has demonstrated that DLP constructs can sustain osteogenic differentiation and promote vascularization within engineered tissues. Printed scaffolds have been shown to support calcium deposition, expression of lineage-specific markers, and the formation of perfusable networks capable of sustaining endothelial growth. These results highlight the potential of DLP bioprinting not only to replicate the structural aspects of native tissues but also to contribute to functional regeneration [36].

Overall, DLP combines high resolution, speed, and precision, making it an increasingly valuable tool in the design of biomimetic scaffolds for regenerative medicine.

**Table 1:** Comparative overview of major bioprinting techniques [34,35,36,37].

Property	Inkjet-Based	Extrusion-Based	Laser-Based	DLP
Material types	Low-viscosity hydrogels enable multicell deposition	Wide range of biocompatible hydrogels, composites, and high cell density	Hydrogels with rapid gelation	Photo-crosslinkable, photosensitive hydrogels
Viscosity range (mPa·s)	3.5–12	30 to >6 × 10^7^	1–300	Generally low-moderate; photocurable inks
Crosslinking mechanism	Chemical and photo-crosslinking	Chemical, photo-crosslinking, shear-thinning, or thermal	Chemical and photo-crosslinking	Photo-crosslinking
Nozzle dynamics	Non-contact nozzle; clogging possible	Nozzle present: shear stress induced during extrusion	Nozzle-free	Nozzle-free
Printer cost	Low	Low-medium	High	Low
Resolution (µm)	100–500	100–500	20–100	20–100
Printing speed	Fast	Slow	Medium	Fast
Cell density	Low	High	Medium	Medium
Cell viability (%)	>85	40–80	Typically, <85	25–90
Advantages	Fast printing speed; low cost	High cell density, versatile materials, good shape fidelity	No nozzle, no clogging; reduced shear stress	High geometric fidelity; rapid fabrication
Drawbacks	Limited to low-viscosity inks; nozzle clogging	Slow speed; shear stress may reduce cell viability	High equipment cost; laser-induced cell damage possible	Limited to photosensitive materials; UV-induced DNA damage risk

## 3. Biomimetic Design Principles

Biomimicry in tissue engineering aims to replicate the complex structures and functions of native biological systems to create scaffolds that support effective tissue regeneration. By emulating the natural extracellular matrix (ECM), biomimetic scaffolds provide cells with biochemical and biophysical cues essential for tissue development and repair [38].

A fundamental aspect of this approach involves mimicking both the structural and functional characteristics of target tissues. For instance, the alignment of fibers in musculoskeletal tissues is crucial for mechanical strength and guiding the cell orientation. Reproducing such features in scaffolds enhances mechanical properties and directs cellular behavior, facilitating tissue-specific regeneration [39].

Native tissues exhibit hierarchical architectures, ranging from nanoscale protein arrangements to macroscale geometries [40]. Advanced imaging techniques such as micro-CT and confocal microscopy enable the capture of these intricate patterns, which can be replicated using fabrication methods such as 3D bioprinting and electrospinning. This replication enhances the scaffold integration and functionality [41].

Designing effective biomimetic scaffolds requires careful consideration of various parameters, including biocompatibility, degradability, and mechanical strength. Features such as the pore size, interconnectivity, and surface topography are critical for supporting nutrient diffusion, vascularization, and cell migration. Functionalization with bioactive molecules can further direct specific cellular responses and accelerate healing [42].

Collectively, these biomimetic design principles provide a comprehensive framework for engineering scaffolds that closely resemble native tissues, promoting improved integration and functionality in tissue engineering applications.

## 4. Bioinks for Bioprinted Scaffolds

When designing scaffolds for tissue engineering, several essential criteria must be addressed to ensure functionality and successful tissue regeneration. These include the biocompatibility, biodegradability, pore interconnectivity, porosity, pore size, and mechanical strength. Biocompatibility ensures that scaffold does not provoke adverse immune responses, while biodegradability allows it to break down into non-toxic byproducts as the tissue regenerates. Importantly, the degradation rate should align with tissue growth to maintain structural support throughout the healing process [43].

The scaffold’s internal architecture is critical. Interconnected pores allow for optimal cell infiltration, nutrient diffusion, and waste elimination. Pore sizes between 150 and 300 μm are typically ideal for bone regeneration, promoting vascularization and cell migration [39]. Mechanical stability is also necessary, particularly for load-bearing tissues, where scaffolds must withstand physiological forces without collapsing [44].

Material choice plays a vital role in scaffold performance. Naturally derived polymers such as collagen, chitosan, and hyaluronic acid offer strong biological cues, enhancing cell adhesion, proliferation, and differentiation [45]. However, they often lack mechanical robustness. Conversely, synthetic polymers such as PCL and PLGA are mechanically stronger, have predictable degradation rates, and are easier to process, yet they are often hydrophobic and less supportive of cellular functions [46].

To overcome these limitations, the use of hybrid scaffolds that combine natural and synthetic polymers has emerged as a promising strategy. These systems aim to integrate the mechanical advantages of synthetic materials with the bioactivity of natural polymers [47]. In bioprinting, parameters such as the bioink viscosity, crosslinking kinetics, and nozzle diameter significantly influence the resulting pore architecture, shape fidelity, and overall reproducibility of scaffolds, parameters that are critical for ensuring both structural stability and favorable biological performance [48].

### 4.1. Classification of Bioinks

Hydrogels are extensively utilized in tissue modeling and regenerative medicine due to their ability to form three-dimensional, hydrophilic polymer networks that swell significantly in aqueous environments. This swelling capacity, resulting from their crosslinked structures, enables hydrogels to mimic the extracellular matrix (ECM), providing a supportive environment for cellular activities [49,50].

In cell culture applications, hydrogels serve as versatile platforms for both two-dimensional (2D) and three-dimensional (3D) cell growth. In the ‘on-gel’ approach, cells are cultured on the surface of the hydrogel, facilitating adhesion and proliferation in a 2D manner. Alternatively, the ‘in-gel’ method supports cell encapsulation within hydrogels, promoting three-dimensional growth that more closely simulates in vivo conditions. The porous nature of hydrogels permits efficient transport of nutrients and waste products, supporting cell viability and function [51,52].

The tunable physicochemical properties of hydrogels, such as their stiffness, degradability, and bioactivity, make them suitable for various biomedical applications, including drug delivery, wound healing, and tissue engineering. By adjusting the composition and crosslinking density, hydrogels can be tailored to meet the specific requirements of different tissues and applications [53].

#### 4.1.1. Natural Polymers

Theoretically, any natural polymer exhibiting a sol–gel phase transition under specific conditions could be utilized for automated layer-by-layer 3D bioprinting, although the practical application is limited. Only a select few natural polymers can be printed at cell-friendly temperatures (e.g., room temperature) without the need for additional physical, chemical, or biological crosslinking agents. This limitation arises because very few natural polymers meet all the essential criteria for 3D bioprinting of cells, tissues, and organs [54].

##### Collagen

Collagen-based scaffolds exhibit excellent biocompatibility, supporting the adhesion, proliferation, and differentiation of osteoblasts, chondroblasts, and mesenchymal stem cells due to their porous structure [54]. Collagen’s use in 3D printing for tissue engineering rests on three major factors. First, scaffolds with gradient morphologies and material compositions enhance functional integration [55]. Second, unlike conventional porous scaffolds, 3D printed collagen scaffolds typically include large, interconnected channels, improving the transport of nutrients, oxygen, and metabolic waste [55]. Third, collagen’s compatibility with living cells allows the creation of both hard and soft tissues, including organ structures. However, the low viscosity and rapid degradation of collagen hydrogels limit their performance as bioinks. Type I collagen remains in a liquid state at low temperatures and transitions into a fibrous structure with increased temperature [55]. These limitations can be mitigated by blending collagen with other polymers such as alginate, gelatin, agarose, or hyaluronic acid to adjust the viscosity and degradation rates. A study reported that combining hyaluronic acid-encapsulated chondrocytes and collagen type I-encapsulated osteocytes yielded promising results for osteochondral tissue bioprinting [56].

##### Alginate

Alginate, a naturally derived polysaccharide, is valued in bioprinting for its affordability, biocompatibility, multiple crosslinking options, and compatibility with various printing methods. However, due to its lower sol–gel transition temperature compared to gelatin, alginate hydrogels are challenging to print independently at ambient conditions (~28 °C). Alginate also exhibits poor cell adhesion relative to other biopolymers [57]. Factors such as the polymer concentration, molecular weight, and cell type greatly influence the viscosity of alginate-based hydrogels. High concentrations may impair cell function post-crosslinking, while lower concentrations promote cell survival and growth [58]. Oxidizing alginate enhances both its degradability and printability, enabling the fabrication of precise lattice structures. Pre-printing optimization of oxidized alginate, especially when loaded with human adipose-derived stem cells (hADSCs), has shown promising results. Studies suggest that alginate formulations with 5% oxidation and 15% concentration offer enhanced osteogenic and chondrogenic potential [59].

##### Gelatin

Gelatin is a partially hydrolyzed derivative of collagen, formed by breaking its triple helix structure into single strands. It is a linear polymer responsive to temperature changes, possessing excellent biocompatibility, rapid biodegradability, high water absorption, and favorable printability. The sol–gel transition for gelatin occurs around 28 °C [60]. To achieve appropriate viscosity for printing, gelatin must be dissolved in solvents such as phosphate-buffered saline or culture media. These gelatin solutions can carry various cells and bioactive compounds (e.g., growth factors, anticoagulants, cryoprotectants) [61]. Upon cooling below 28 °C, the gelatin undergoes sol–gel transition—physically crosslinking to form hydrogels that encapsulate cells or bioactive agents. Gelatin can be used alone or with natural polymers such as alginate, chitosan, collagen, fibrinogen, hyaluronic acid, agar, and Matrigel to create multifunctional bioinks [62,63].

Kang et al. developed a bioink combining phenol-rich gelatin (GHPA) with graphene oxide (GO) capable of inducing myogenesis through dual enzyme-mediated crosslinking [64]. This GO/GHPA bioink maintained gelatin’s proteolytic degradability even after GO inclusion and phenol modification, which is critical for regulating cellular behavior. Similarly, a GelMA/HAMA-based bioink was used to fabricate 3D-printed skin constructs with features such as hair follicle structures and a layered dermal–epidermal morphology [65]. This formulation exhibited optimal printability, cytocompatibility, and structural stability.

In another study, Jo et al. incorporated MXene nanoparticles into GelMA/HAMA-based bioinks (termed GHM) to promote myogenesis [66]. These composite hydrogels demonstrated excellent microporosity, biocompatibility, and print fidelity, and effectively supported skeletal muscle cell differentiation without external cues [66].

##### Hyaluronic Acid (HA)

Hyaluronic acid (HA), or hyaluronan, is a linear polysaccharide composed of D-glucuronic acid and N-acetyl-D-glucosamine. Found naturally in the extracellular matrix, HA supports cell proliferation, angiogenesis, and receptor-mediated interactions due to its high biocompatibility and degradability. It is enzymatically degraded by hyaluronidase, β-glucuronidase, and N-acetyl-glucosaminidase into lower molecular weight forms. HA forms highly viscous hydrogels at low concentrations, making it an excellent thickening agent for gelatin-based bioinks [67]. However, like many natural polymers, HA has limited mechanical strength and shape retention in 3D bioprinting. To improve these characteristics, HA is often chemically or physically crosslinked with other materials. For example, methacrylated HA is frequently blended with gelatin methacrylate (GelMA), mimicking the glycosaminoglycan and collagen composition of natural skin [68].

##### Chitosan

Chitosan, a natural polysaccharide derived from the deacetylation of chitin (commonly found in shrimp shells), is widely applied in tissue engineering due to its biodegradability, antibacterial activity, and low toxicity. Chitin is composed of D-glucosamine and N-acetyl-D-glucosamine units linked via glycosidic bonds, and is abundantly found in crustaceans (up to 30%), as well as in certain algae and fungi. Recent advancements have led to the development of chitosan-based hydrogel composites incorporating gelatin, alginate, and hydroxyapatite for bone tissue regeneration [69].

#### 4.1.2. Synthetic Polymers

##### Poloxamers

Poloxamers are non-ionic triblock copolymers composed of hydrophilic poly(ethylene oxide) (PEO) segments flanking a central hydrophobic poly(propylene oxide) (PPO) chain. This class of polymers was first patented by Irving Schmolka of BASF in 1973. They are commercially available under trade names such as Pluronic F68 and Pluronic F127. Among these, Pluronic F68 has been shown to enhance mechanical strength and stiffness. These polymers, often referred to as poloxamers or pluronics, are FDA-approved due to their non-toxic composition. In aqueous environments, they exhibit a sol-to-gel transition when the temperature surpasses a specific lower critical gelation temperature (LCGT) [70].

In bioprinting and scaffold fabrication, Pluronic F127 is frequently used as a mold or sacrificial material due to its facile micellar-packing gelation mechanism. However, its inherent mechanical weakness and susceptibility to dissolution in aqueous environments limit its structural application unless chemically modified. Functionalization with photo-crosslinkable acrylate groups can enhance its mechanical strength, although its lack of intrinsic bioactivity or cell adhesion sites remains a challenge [70].

##### Polylactic-Co-Glycolic Acid (PLGA)

PLGA is a biodegradable and cytocompatible copolymer widely used in biomedical applications. It exhibits mechanical properties like human cortical bone and has osteoconductive potential. However, its hydrophobic nature presents limitations for tissue engineering applications. Additionally, its linear structure leads to relatively low mechanical stiffness and rapid degradation. These shortcomings can be partially addressed via blending with other polymers such as polycaprolactone (PCL), which improves its structural integrity and reduces polymer fragmentation that may otherwise cause undesired immune responses [71].

##### Polyvinyl Alcohol (PVA)

PVA is a synthetic polymer derived from the polymerization of vinyl alcohol and acetate. It possesses a semi-crystalline structure, offering characteristics such as biodegradability, biocompatibility, and chemical stability. Its mechanical strength is comparable to that of natural cartilage, and it supports the development of complex structures suitable for bone tissue engineering. PVA’s hydrophilic nature allows it to maintain functionality under a wide range of pH and temperature conditions. Due to the hydroxyl (–OH) groups along its backbone, PVA is highly water-attracting and easily modifiable or blendable with other polymers, which improves its rheological properties and printability in extrusion-based bioprinting. PVA hydrogels also exhibit shear-thinning behavior, enabling smooth nozzle flow and stable 3D constructs. Despite these advantages, its water solubility complicates the control over swelling behavior, which can affect its long-term structural stability in aqueous environments [72].

##### Polycaprolactone (PCL)

PCL is a cost-effective polymer known for its biocompatibility, rigidity, and degradability, making it suitable for bioink and scaffold applications. It exhibits excellent stability, with a degradation profile lasting up to several months and a biological half-life of around three years. Its porous and rough surface structure enhances cell attachment and supports osteoconductive functions in engineered scaffolds. However, its slow degradation rate can hinder timely tissue regeneration, and its hydrophobic character contributes to poor bioactivity, resulting in reduced cell proliferation and suboptimal tissue integration [73,74].

##### Polylactic Acid (PLA)

PLA is a popular biodegradable polymer with tunable chemical and physical properties. It is synthesized from lactic acid monomers, which can be polymerized in different stereochemical configurations using L- or D-lactic acid. PLA variants containing D-lactic acid are more hydrophilic and susceptible to hydrolytic cleavage due to their amorphous structures. This makes PLA suitable for applications requiring material resorption within 8 to 12 months. PLA’s adaptability, processability, and degradation profile have led to its use in bone tissue engineering. Advances in 3D printing have facilitated the development of PLA-based composites, such as those integrated with graphene oxide (GO), to improve the osteoconductive potential. The incorporation of GO enhances mechanical and biological properties, making these composite materials promising for guided bone regeneration (GBR) and potentially other tissue-engineering applications [75].

While Table 2 summarizes the characteristics of natural, synthetic, and hybrid bioinks, achieving an optimal balance of rheological and mechanical properties is critical for scaffold performance. Modifying the composition or using crosslinking can improve the shape fidelity and mechanical stability but may compromise cell viability, migration, or proliferation. Strategies such as dual-step or tunable crosslinking, shear-thinning and self-healing hydrogels, and incorporation of nanomaterials (e.g., graphene oxide, carbon nanotubes) help maintain both structural integrity and cytocompatibility. Granular hydrogels enhance the porosity and nutrient transport, while sacrificial inks and support baths enable complex architectures without affecting cell survival. Advanced approaches, including stimuli-responsive 4D bioinks and machine learning-guided formulations, allow predictive optimization of the printability, mechanics, and biological performance. Together, these approaches demonstrate that careful tuning of the material composition, crosslinking, and rheology is essential to fabricate scaffolds that are mechanically robust while supporting cell proliferation, differentiation, and tissue development [50].

## 5. Applications of Bioprinted Biomimetic Scaffolds

### 5.1. Skin Tissue Engineering

To replicate the layered architecture of native skin, researchers have employed a layer-by-layer bioprinting approach to fabricate multi-layered synthetic tissue constructs. In this method, fibroblasts are deposited in the second layer, while keratinocytes are introduced separately in the eighth layer. Notably, high cell viability has been observed on a poly(dimethylsiloxane) (PDMS) mold featuring three-dimensional surface structures, demonstrating promising printability for applications such as skin wound healing. In a related study, a similar bioprinting platform was utilized to print keratinocytes, fibroblasts, and collagen, employing eight separate nozzles to reconstruct the epidermis, dermis, and dermal matrix layers of human skin. Immunofluorescence staining and histological evaluations confirmed that the bioprinted skin tissue exhibited both structural and biological characteristics comparable to those of native human skin. The advantages of 3D bioprinting in this context include precise shape retention, design flexibility, reproducibility, and scalability for high-throughput culture. These findings position such constructs as valuable models for studying the pathophysiology of skin-related diseases [76].

Researchers developed collagen scaffolds composed of parallel collagen fibers arranged in sequential layers, exhibiting an exceptionally high porosity (>95%). Following sub-zero processing at −76 °C to solidify the constructs, keratinocyte–fibroblast co-cultures were seeded onto the printed scaffolds to assess cellular proliferation, migration, and differentiation. Building on this work, the same research group fabricated collagen–alginate composite scaffolds using a coaxial bioprinting method. This approach significantly enhanced the mechanical properties, increasing the scaffold’s modulus by approximately sevenfold compared to the earlier version [77].

Wu et al. developed a curvilinear printable bioink composed of gelatin and polyurethane, embedded with keratinocytes, fibroblasts, and endothelial progenitor cells. This customized bioprinted construct was designed using the specific wound contours of individual rodents and implanted in a chronic, irregular wound model. After 28 days, the implant promoted complete re-epithelialization, dermal regeneration, significant neovascularization, and enhanced collagen formation [78]. Zhang et al. established a quick and straightforward approach to formulate a bioink using microfragmented adipose extracellular matrix (mFAECM), overcoming the prior challenge of adipose tissue’s incompatibility with 3D bioprinting. The in vitro results confirmed the effectiveness of the mFAECM bioink in enhancing wound healing by stimulating collagen secretion, tissue remodeling, and the formation of new blood vessels [79].

Li et al. had created an innovative 3D-bioprinted hydrogel embedded with methylene blue nanoparticles to enable photodynamic therapy-based antibacterial activity, which was evaluated in vitro. The study revealed a notable decrease in the viability of common wound-associated pathogens. These findings highlight the broader utility of 3D bioprinting, not only for fabricating skin substitutes but also for advancing wound dressings and infection management strategies [80].

Bioprinting has shown significant translational potential in skin regeneration, moving from animal studies to early clinical applications. In situ handheld bioprinters capable of depositing autologous keratinocytes and fibroblasts directly into wounds have accelerated healing and reduced scarring in porcine burn models [81]. Full-thickness human skin equivalents containing epidermal, dermal, and hypodermal layers have also been successfully grafted in large animals, demonstrating rapid vascularization, enhanced collagen deposition, and diminished fibrosis. Importantly, clinical translation is underway; for instance, the dermo-epidermal substitute Poieskin^®^ has entered first-in-human evaluations as a potential alternative to autologous grafts for full-thickness wounds [82]. These advances highlight the feasibility of bioprinted skin scaffolds while emphasizing the need for larger trials to establish long-term safety and efficacy [83].

### 5.2. Bone Tissue Engineering

A widely utilized strategy in bone tissue engineering is 3D bioprinting for the fabrication of scaffolds. Over the last decade, researchers have created PCL-HA (polycaprolactone–hydroxyapatite) scaffolds based on CT-derived 3D reconstruction data using the fused deposition modeling (FDM) technique. These scaffolds have been further evaluated for their ability to withstand biologically relevant mechanical loads. A combination of PCL, poly(lactic-co-glycolic acid) (PLGA), β-tricalcium phosphate (β-TCP), and a mineralized extracellular matrix (ECM) synthesized by human nasal inferior turbinate-derived mesenchymal stromal cells (hTMSCs) was used to construct scaffolds. The presence of the ECM endowed these constructs with both osteoinductive and osteoconductive properties, supporting mineralization and bone tissue mimicry [84].

Another technique developed for bone tissue bioprinting involved the alternating deposition of bioink and PCL fibers on a gamma-irradiated alginate base. This method employed adult mesenchymal stem cells (MSCs) functionalized with arg-gly-asp (RGD) adhesion peptides to improve chondrogenic differentiation, while the PCL fibers contributed to enhanced mechanical strength. The strategic placement of bioink between every second PCL layer created a network of bioink-free interconnected channels, which facilitated improved nutrient transport within the construct. Kang et al. introduced an integrated tissue–organ printing system (ITOP) that utilized extrusion-based printing to pattern cell-laden hydrogels and biodegradable polymers alongside sacrificial hydrogels such as Pluronic F-127. This approach enabled the formation of internal microchannels within the printed constructs and was applied to fabricate various tissue types, including bone and cartilage [85].

Beyond preclinical advances, several translational studies have demonstrated the feasibility of bioprinted scaffolds in bone regeneration. A pilot clinical application of patient-specific, 3D-printed calcium phosphate scaffolds for craniofacial reconstruction showed encouraging bone integration and functional outcomes. In situ bioprinting approaches are also emerging, where bioinks carrying stem cells or growth factors can be directly deposited into bone defects during surgery to accelerate repair and reduce secondary grafting. More recently, a case report described the successful implantation of a customized PCL/β-TCP scaffold loaded with platelet-rich plasma to repair a tibial defect, marking one of the first clinical uses of a biologically active bioprinted bone construct. While large-scale clinical trials remain limited, these examples underscore the translational potential of bioprinted bone scaffolds and their gradual move toward clinical application. While these reports are encouraging, larger controlled trials are still needed to confirm the safety, long-term function, and reproducibility before routine clinical adoption [86,87,88].

### 5.3. Cardiovascular Tissue Engineering

Cardiovascular diseases (CVDs) remain a leading cause of death worldwide, particularly in developed countries. Each year, more than eight million cases of myocardial infarction are reported globally. In addition to heart attacks, conditions such as valve stenosis also impact cardiac function. A major concern in these disorders is the loss of cardiomyocytes, which lack the capacity for self-repair or regeneration [89]. Tissue engineering offers promising alternatives for addressing damage to cardiac tissues, such as blood vessels and heart valves.

Traditional approaches in cardiovascular tissue engineering rely on biomaterial scaffolds that support the growth, proliferation, and differentiation of stem cells. Both synthetic and natural hydrogels, along with decellularized tissue matrices, have been explored due to their biocompatibility and close resemblance to native extracellular matrices. Autologous and allogenic stem cells are favored in cardiac applications due to their broad availability and minimal risk of immune rejection. Nevertheless, engineering a fully functional cardiac tissue construct remains highly complex, as it requires the integration of multiple cell types, including cardiomyocytes, endothelial cells, and fibroblasts. Achieving spontaneous, synchronized myocardial contraction adds another layer of difficulty [90].

**Figure 2:**
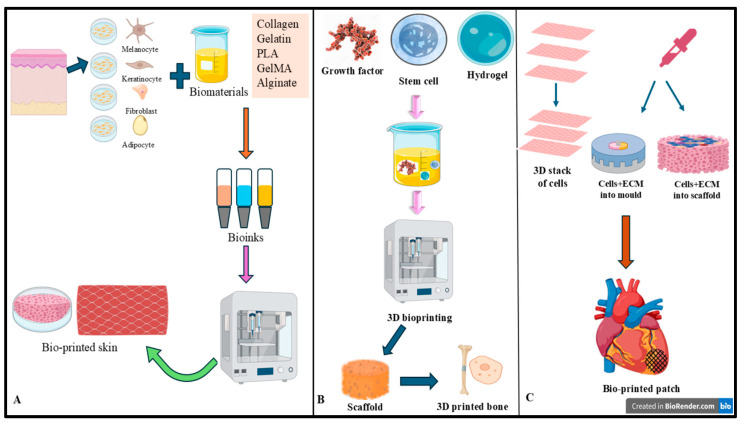
The 3D bioprinting of (**A**) skin, (**B**) bone, and (**C**) heart tissues.

Figure 2 illustrates the workflow and applications of 3D bioprinting, demonstrating its use in generating bioprinted skin constructs, bone scaffolds, and cardiac patches.

To address these challenges, 3D bioprinting presents a valuable solution. It enables the precise, layer-by-layer construction of functional cardiac tissues. Several studies have investigated the fabrication of biomaterial-based scaffolds and tissue-on-a-chip platforms aimed at replicating the myocardial structure and function using 3D printing technologies [91].

### 5.4. Neural Tissue Modeling

Regenerating damaged neural tissues caused by traumatic brain or spinal cord injuries, as well as neurological disorders such as stroke, Alzheimer’s disease, Parkinson’s disease, multiple sclerosis, and Huntington’s disease, remains one of the most complex clinical challenges. One promising approach involves the fabrication of 3D nerve models that closely resemble the native extracellular matrix (ECM) [92].

Effective neural models must meet several essential criteria including electroconductivity, appropriate elastic properties, hierarchical microarchitecture, and neurocompatibility to support nerve cell adhesion and proliferation. Among various 3D bioprinting techniques, extrusion-based bioprinting (EBB) stands out for its ability to process a broad range of materials, such as cell suspensions, cell-laden hydrogels, solutions, thermoplastics, thermosets, and elastomers, making it suitable for neural tissue fabrication. However, the limited availability of neural-specific bioinks that accurately replicate the biochemical and mechanical characteristics of the neural ECM has restricted their application in brain tissue engineering [93].

To address the above limitation, a filler-free bioink was developed utilizing a thiol/catechol chemistry approach, in which thiolated Pluronic F-127 was crosslinked with dopamine-conjugated gelatin and dopamine-conjugated hyaluronic acid (HA) [94]. In another study, researchers created a biodegradable polyurethane-based hydrogel that is responsive to dual stimuli, aiming to optimize both mechanical performance and cellular compatibility. These hydrogels featured relatively low viscosity, helping to reduce excessive shear stress and minimize the risk of nozzle clogging during extrusion. They also exhibited sufficient shear yield strength and structural integrity, allowing them to support their own weight without deformation during layer stacking [78].

Furthermore, neural stem cells (NSCs) demonstrated the ability to adhere, proliferate, and differentiate into neural lineages on these printed constructs. A portable, bathless 3D printing system was also utilized to fabricate brain-like architectures composed of multiple layers of neural cells encapsulated in gellan gum hydrogels modified with the arginine–glycine–aspartate (RGD) peptide. The RGD-functionalized hydrogels showed enhanced cell viability and neural network formation compared to unmodified gellan gum [92]. These studies collectively illustrate advancements in neural tissue bioprinting through the development of innovative biomaterials and bioinks that foster neural cell function and structural fidelity.

### 5.5. Hepatic Tissue Modeling

Hepatic lobules are the fundamental structural and functional components of liver tissue. Due to their small size and intricate architecture, recreating hepatic lobules in vitro poses significant challenges for tissue engineering. To overcome these obstacles, high-resolution 3D bioprinting techniques such as digital light stereolithography have been employed. For instance, this technology has enabled the fabrication of branched biliary epithelial structures, mimicking the bile duct networks of the liver [95]. Additionally, it has facilitated the creation of microscale hexagonal units incorporating hepatocytes, endothelial cells, and adipose-derived stem cells to replicate the native liver lobule architecture [96].

Beyond digital light stereolithography, the use of precursor cartridges has emerged as an effective strategy for enhanced printing resolution. These cartridges are shaped to match the desired tissue architecture and can be loaded with various bioinks in separate compartments. When combined with microfluidic systems, this approach enables the fabrication of liver lobule-like microstructures with a resolution of up to 20 µm, significantly finer than traditional 3D bioprinting methods. This microscale-to-macroscale fabrication strategy can be integrated with extrusion-based bioprinting, offering considerable potential for future applications. Nevertheless, a major limitation of high-resolution printing techniques is their reduced throughput, presenting a tradeoff between precision and scalability that must be balanced according to specific research goals [97].

The design of the bioprinted model and the selection of cell types are critical for accurately simulating the liver’s physiological environment. Because the liver comprises various cell types, including hepatocytes, endothelial cells, Kupffer cells, hepatic stellate cells, and progenitor cells, a multicellular approach is necessary. Thus, 3D bioprinting enables the construction of complex, perfusable in vitro liver models. One notable example is a multicellular perfusable structure developed with a microfluidic system, where the central lumen was created using a sacrificial bioink. GelMA and fibrin-based bioinks loaded with HepG2 and other cell types were used to mimic liver functionality, while a poly(dimethylsiloxane) (PDMS) outer layer provided mechanical strength and integration capability with vascular or microfluidic components. Such constructs demonstrate the potential of 3D-bioprinted tissues to serve as vascularized liver substitutes in regenerative applications [98].

The cell distribution is also essential for replicating the liver’s complex functionality. Adjusting the ratios and spatial arrangement of different cell types enhances the biological relevance of the printed tissue. Furthermore, 3D-bioprinted liver models have been employed in drug screening, offering an alternative to animal testing and supporting more accurate disease modeling. One major application includes testing anti-cancer therapies. For instance, tumor models created with cells of intrahepatic cholangiocarcinoma have shown promise in drug efficacy studies. These bioprinted tumor models are poised to become vital tools for personalized medicine, helping guide clinical treatment decisions [99].

In summary, 3D-bioprinted liver constructs offer a biomimetic platform for studying liver physiology, modeling disease mechanisms, and exploring therapeutic strategies. They also hold potential as graft sources for regenerative medicine and tools for drug discovery and personalized treatment planning. Additionally, integrating advanced analytical methods with post-printing processes will be crucial to furthering research in liver biology and pathology. Overall, continued development and refinement of 3D bioprinting technologies and biofabrication strategies are essential for replicating the complex liver microenvironment and enhancing clinical translation [100].

### 5.6. Lung and Tracheal Tissue Modeling

The primary function of the lungs is gas exchange, which occurs in the alveoli through the air–blood barrier. The complexity and small size of these structures (~200 μm for alveoli and 0.62 μm for the air–blood barrier) make 3D bioprinting of alveolar models a challenge. Horvath et al. (2015) printed a gelatinous protein mixture (Matrigel™) with endothelial and epithelial cells, achieving thinner and more uniform cell layers than manual methods, although the barrier is not yet suitable for clinical applications [101]. Grigoryan et al. (2019) used polyethylene glycol diacrylate to print a distal lung model with vascular and airway spaces and demonstrated oxygen transport via human red blood cells [102]. However, the airway space was in millimeter dimensions and lacked cells, requiring further miniaturization to mimic real alveolar structures [101,102].

For tracheal grafts, 3D bioprinting often uses a combination of synthetic polymers such as PCL and PLA, as well as natural polymers such as collagen or alginate, which are seeded with cells such as chondrocytes or epithelial cells. PCL, a biocompatible and biodegradable polymer, is commonly used for temporary applications such as tracheal grafts, as it takes years to degrade. When combined with hydrogel matrices and seeded with cells, PCL scaffolds improve tissue integration. Studies have shown that PCL-based grafts with chondrocytes promote cartilage formation and epithelial regeneration in vivo. PLA and its copolymer PLCL also show promise, with PLA degrading in 12–16 months, while PLCL degrades in 12–24 months, offering suitable mechanical properties for tracheal grafts. Additionally, non-biodegradable materials such as polyurethane (PU) have been explored for long-term grafts, although further research is needed to confirm their long-term suitability [103,104].

Mesenchymal stem cells (MSCs) are often used to enhance tissue maturation. When seeded onto scaffolds, MSCs improve tissue integration, as seen in studies where MSC-seeded grafts demonstrated successful integration with the host trachea, promoting epithelial regeneration and neo-cartilage formation [105].

## 6. Organoids and Organ-on-a-Chip Platforms

**Figure 3:**
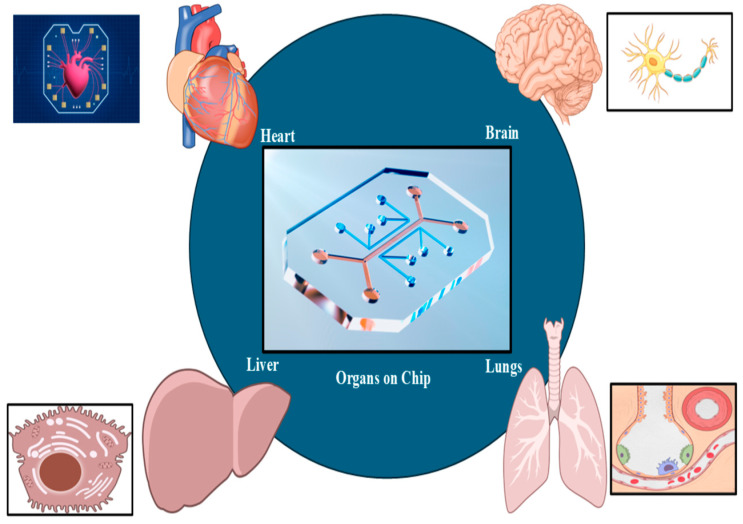
Organs on a chip.

Organ-on-a-chip (OOC) technology (Figure 3) allows for precise fluid manipulation within microfluidic chips, simulating the physiological, chemical, and mechanical properties of tissues. This makes OOC a promising tool for in vitro drug screening and physiological modeling. In recent years, the rapid progress of this technology has been driven by innovations in three-dimensional (3D) printing techniques. Thus, 3D printing not only enables the fabrication of microfluidic chips using materials such as resins and polydimethylsiloxane but also allows the creation of biomimetic tissues with bioinks, such as cell-loaded hydrogels [106].

Heart-on-a-chip models have been widely studied to replicate cardiac tissue microenvironments and measure contractility and electrophysiology. Zhang et al. used direct ink writing (DIW) bioprinting to create a 3D endothelial bed with microfibrous hydrogel scaffolds, seeding cardiomyocytes to form an aligned myocardium capable of spontaneous contractions [107]. These organoids were then placed in a microfluidic perfusion bioreactor for cardiovascular toxicity evaluation [107]. In another study, cardiac microphysiological devices were developed using multi-material DIW printing with sensors, enabling non-invasive monitoring of drug responses and contractile stresses [108]. In a study, TPP 3D printing and soft lithography were used to engineer cardiac microtissues on a microfluidic platform, incorporating strain actuators and force sensors to study tissue responses under mechanical loading [109]. Some researchers employed multimaterial DIW printing to fabricate elastic membranes with optical waveguides, electrodes, and microfluidics for recording cardiomyocyte field potentials [110].

Vessel-on-a-chip models are crucial for studying biological molecule delivery, microscale fluid dynamics, and intercellular communication in 3D extracellular matrix environments. In a study, coaxial extrusion bioprinting was used to fabricate vascular structures with layered arrangements of smooth muscle and endothelial cells, resulting in constructs that demonstrated good tissue integration, mechanical strength, and functional vaso-activity [111]. In another approach, inkjet bioprinting was employed to produce millimeter-scale vascular channels, which later developed capillary-like networks through natural maturation processes [112]. Additionally, a direct ink writing (DIW)-based sacrificial bioprinting technique was utilized to create a thrombosis-on-a-chip model, where bifurcated microchannels lined with endothelial cells were perfused with whole blood, leading to the formation of thrombi [113]. These strategies highlight the potential of bioprinting to replicate complex vascular systems for use in tissue engineering and disease modeling. This platform has been used to explore fibrosis and other vascularized fibrotic disease models [114].

The nervous system, comprising the brain, spinal cord, and nerves, transmits messages between the brain and body. Diseases such as stroke, Parkinson’s, and Alzheimer’s can cause severe pain. The blood–brain barrier (BBB) protects the brain, making drug delivery challenging. To mimic the BBB, brain-on-a-chip systems have been developed. A vascularized neural network to identify BBB-penetrating drugs was developed using a microfluidic system with a polycaprolactone/poly (d,l-lactide-co-glycolide) vasculature [115]. Johnson et al. used ME 3D bioprinting to create a nervous system-on-a-chip for studying viral infections, showing that Schwann cells resist infection [116]. Salmon et al. developed SLA 3D-printed chips with a coculture system, where vascular cells interacted with a cerebral organoid, forming an integrated neurovascular system [117]. These advances in brain-on-a-chip models help to simulate the BBB and study neurological diseases and drug delivery.

The liver plays a crucial role in detoxifying blood, regulating blood sugar, and controlling blood clotting. Liver-on-a-chip models have become essential for simulating liver functions and developing therapies for liver diseases. In a study, a 3D perfusable liver chip was developed using hiPSC-derived organoids for long-term culture. Extrusion bioprinting was utilized to fabricate hepatic lobular arrays with multicellular and multimaterial structures, where endothelial cells were arranged around hepatic cells, leading to improved albumin and urea secretion. Additionally, a tri-culture model was constructed using digital light processing (DLP) printing with hiPSC-derived hepatic progenitor cells, human endothelial cells, and adipose-derived stem cells, which demonstrated enhanced liver-specific functions [118]. Lee et al. created a liver-on-a-chip with vascular and biliary systems, improving drug responses and liver-specific gene expression compared to models without biliary channels. These advancements demonstrate the potential of liver-on-a-chip models for drug testing and liver disease research [119].

## 7. Advanced Technologies and Innovations

Over the past decade, 4D printing has evolved as an extension of 3D printing, introducing time as the fourth dimension. Initially proposed in 2013 by Tibbitts from MIT’s Self-Assembly Lab, 4D printing was described as “3D printing + time,” allowing printed structures to transform their shape over time. Today, it is defined as the fabrication of 3D constructs that respond to external stimuli such as heat, pH, water, light, and magnetic or electric fields. This transformation is made possible through the integration of smart materials, those that change properties in response to stimuli, and intelligent design strategies that enable pre-programmed, time-dependent behavior. As a result, 4D printing has gained attention in the biomedical field for creating dynamic devices and tissue constructs [120].

The emergence of 4D bioprinting, which builds upon 4D printing principles using biocompatible smart materials, bioactive molecules, and living cells, allows for the fabrication of dynamic living tissues. Unlike traditional 3D bioprinting, which often results in static structures, 4D bioprinting enables constructs that mimic the dynamic remodeling of native tissues. However, the definition of 4D bioprinting remains debated. Some researchers argue that 3D bioprinting is inherently 4D, given that printed constructs undergo cellular reorganization and material degradation over time. Others suggest that true 4D bioprinting must involve predictable, externally triggered transformations. To clarify, 4D bioprinting can be defined as the layer-by-layer deposition of smart bioinks, designed through pre-programmed patterns, to produce dynamic, stimuli-responsive tissue constructs [121].

**Figure 4:**
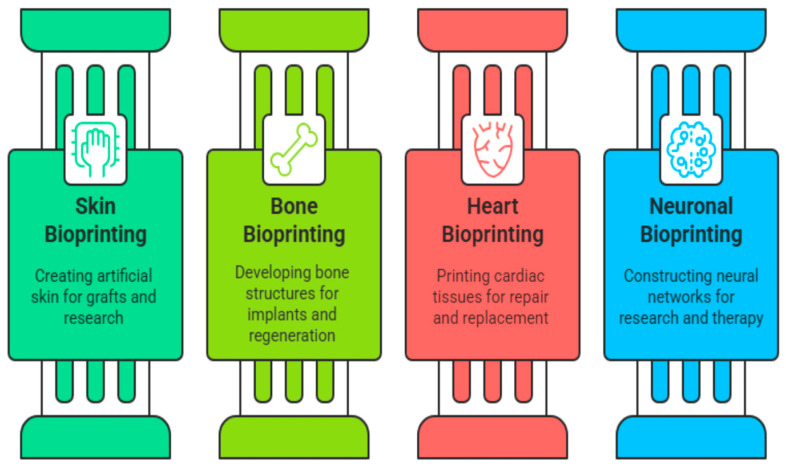
Application of 4D bioprinting.

The advent of 4D bioprinting has introduced a promising approach for creating dynamic, living constructs that closely replicate the functional behavior of native tissues and organs. In recent years, this technology has seen growing use in the fabrication of responsive biological structures such as skin, bone, cartilage, and more as shown in Figure 4 [121].

### 7.1. 4D Bioprinting for Skin, Bone, and Other Tissue Engineering

The emergence of 4D bioprinting has revolutionized tissue engineering by enabling the fabrication of dynamic living constructs that better mimic the physiological behavior of native tissues and organs [122]. Although 3D bioprinting has enabled the creation of cell-laden skin substitutes that enhance skin regeneration, its static nature often results in geometric mismatches with irregular wounds, impeding tissue integration. More recently, 4D bioprinting has demonstrated the ability to create dynamic skin grafts that adapt their shape to wound geometries, enhancing integration and accelerating healing. Additionally, 4D-printed in vitro skin models have been developed using LAB-patterned myofibroblasts on collagen matrices to study skin cell–ECM dynamics. These constructs exhibited anisotropic collagen remodeling driven by fibroblast-mediated traction forces, providing valuable insight into wound healing mechanisms [123].

Bone tissue engineering has also greatly benefited from 4D bioprinting, primarily using shape memory hydrogels (SMHs) and shape memory polymers (SMPs). Two major approaches are employed: one involves injectable SMH inks that solidify under physiological conditions, while the other relies on hydrogel constructs with crosslinking gradients that induce controlled folding. For example, photo-crosslinkable hydrogels such as PNIPAm, gelatin, and collagen have been employed to create cell-laden constructs that exhibit shape transformation. Mesenchymal stem cells (MSCs) embedded in these hydrogels retained high viability, proliferated robustly, and demonstrated osteogenic differentiation and mineralization, highlighting their potential for bone regeneration [124].

Beyond skin and bone, 4D bioprinting has been applied to fabricate other tissues, including muscle and cardiac tissues. Myoblast-laden self-scrolling constructs created through a combination of melt electrowriting (MEW) and extrusion printing have demonstrated high cell viability and alignment, essential for muscle regeneration. Similarly, thin hydrogel membranes produced from oppositely charged polymers such as GelMA and PLL have shown promise in regenerating delicate tissues such as the cornea and epidermis. Moreover, bilayer hydrogels embedded with fibroblasts and magnetic particles have enabled cyclic folding movements, simulating the contractile behavior of native tissues and offering platforms to study biomechanical cell responses [125].

Cardiac tissue engineering has seen the development of 4D-printed shape-morphing patches from materials such as graphene–epoxy composites and PLA, capable of matching the heart’s curvature and promoting cardiomyocyte maturation. Additionally, ventricles printed using fiber-infused gelatin–alginate inks exhibited spontaneous contraction, mimicking the native myocardium [126]. Despite these advancements, the fabrication of fully functional solid organs such as the liver or kidneys remains a significant challenge due to the complexity of their cellular diversity and vascular networks.

Overall, 4D bioprinting holds tremendous promise for regenerative medicine by enabling the creation of responsive, biomimetic tissues with dynamic functionality.

### 7.2. Artificial Intelligence Augmented Scaffold Design

Artificial intelligence (AI) is increasingly being used in scaffold design and bioprinting to improve both accuracy and efficiency. By combining computational models with biological data, AI can predict the scaffold strength, refine pore structures, and generate patient-specific designs that closely resemble native tissues. This integration of modeling and biology has created new opportunities for scaffolds that are both structurally stable and biologically functional [127].

Applications of AI have been reported across several tissues. In bone regeneration, AI has been applied to optimize the scaffold geometry and material distribution, leading to improved strength and mineralization. For cartilage, AI-assisted design has enabled layered architectures that reproduce the natural gradient of tissues. In skin repair, AI models have been used to create microchannel networks that support vascularization and wound healing. Neural tissue engineering has also benefited from machine learning approaches that design aligned structures to guide axonal growth and improve connectivity [128].

In addition to architectural design, AI supports process optimization by adjusting printing parameters such as the extrusion pressure, nozzle speed, and temperature. When integrated with imaging systems, AI can provide real-time feedback, reducing errors and improving the reproducibility. Recent developments have also explored the use of generative AI to design scaffold architectures beyond conventional CAD models, enabling the fabrication of complex and multi-material structures [129].

Despite these advances, several challenges remain. Large, high-quality datasets are required to train reliable models, biological outcomes remain difficult to predict, and AI-generated decisions are not always easy to interpret [129]. Addressing these issues through explainable models, better data, and interdisciplinary collaboration will be essential for translating AI-enabled bioprinting into safe and clinically acceptable applications.

## 8. Regulatory, Ethical, and Translational Perspectives

Recently, 3D printing technology has made remarkable strides in the medical, pharmaceutical, and healthcare sectors, with regulatory bodies such as the U.S. Food and Drug Administration (FDA), the European Medicines Agency (EMA), and the National Medical Products Administration (NMPA) playing increasingly proactive roles in overseeing its safe implementation. Several significant milestones have been achieved, including the FDA’s clearance in 2013 of the OsteoFab^®^ Patient-Specific Cranial Device, the first 3D-printed cranial implant developed by Oxford Performance Materials. In 2016, the CASCADIA™ Lateral Interbody System, a titanium-based 3D-printed spinal implant developed by K2M Group Holdings (later acquired by Stryker), received both FDA 510(k) clearance and CE marking. Since then, several 3D-printed medical devices have been cleared through the FDA’s 510(k) process, with numerous studies validating their successful use in clinical settings [126].

Despite this progress, no FDA-approved or cleared 3D bioprinted tissues or organs currently exist. The primary roadblock lies in the lack of standardization across 3D bioprinting platforms, encompassing printing technologies, bioinks, cell sources, and fabrication protocols. Unlike conventional 3D-printed medical devices, bioprinted constructs incorporate living cells and complex tissue architectures, which pose significant challenges to regulatory assessment. While good manufacturing practices, such as documenting the origin of cells, verifying cell viability and functionality, and ensuring sterility, can be implemented, unresolved issues persist. These include intricate logistics and the absence of long-term safety data for implanted bioprinted tissues in human recipients.

Moreover, only a limited number of facilities worldwide possess the infrastructure and technical capability to fabricate bioprinted tissues or organs. Typically, patient-derived cells and extracellular matrices must be transported to specialized biofabrication centers, where the constructs are printed and matured under controlled conditions before being returned to the clinic for transplantation. This creates substantial logistical hurdles, as living tissue constructs require meticulous coordination, rapid transport, and strict environmental control to preserve viability and functionality.

However, recent clinical breakthroughs offer promising indications of bioprinting’s translational potential. A landmark case occurred in June 2022 when 3D Bio Therapeutics successfully conducted the first human implantation of an autologous bioprinted ear, marking a significant milestone in regenerative medicine and personalized therapy.

On the pharmaceutical front, 3D printing has also begun to reshape drug development and manufacturing. In 2013, the FDA approved an Investigational New Drug (IND) application for Spritam^®^, the first 3D-printed medication. Its eventual clearance in 2015 was greatly facilitated by the FDA’s Emerging Technology Team (ETT), which was established in 2014 to support innovative pharmaceutical technologies. Recognizing the potential of 3D printing, the FDA officially identified it in 2017 as a transformative platform for drug manufacturing.

In 2020, Triastek joined the FDA’s Emerging Technologies Program with its melt extrusion deposition (MED) 3D printing platform, which was accepted as a valid regulatory pathway. The company’s T19 formulation—only the second 3D-printed drug product globally—was cleared in January 2021. Triastek has also engaged in international regulatory and scientific discussions, including its participation in the Q13 Continuous Manufacturing Conference hosted by China’s CDE, demonstrating global momentum in 3D printing-based drug innovation [126].

In 2021, the FDA’s Center for Drug Evaluation and Research (CDER) tasked the National Academies of Sciences, Engineering, and Medicine (NASEM) with evaluating the future of pharmaceutical manufacturing. The resulting report projected that 3D printing would progressively replace conventional manufacturing techniques, given its capacity for on-demand, patient-specific drug production. However, the report also underscored a crucial gap: the lack of universally accepted regulatory guidelines specific to 3D-printed drug products, highlighting the need to establish baseline standards [126].

While 3D printed medical devices have received regulatory clearance and pharmaceutical applications are gaining momentum, 3D bioprinted tissues and organs remain at the frontier of innovation, awaiting breakthroughs not only in technology but also in regulatory frameworks and infrastructure. The evolution of this field hinges on continued collaboration between scientists, clinicians, engineers, and regulators to address the inherent complexities of bioprinted constructs and usher them safely into clinical use.

## 9. Challenges and Future Perspectives

Despite its transformative potential, 3D printing in pharmaceuticals and bioprinting faces several research challenges that must be addressed for clinical and industrial translation. A major bottleneck is the limited range of printable and biocompatible materials. While polymers used in fused deposition modeling (FDM) or stereolithography (SLA) offer good printability, many are unsuitable for heat-sensitive or biologically active compounds, restricting the development of patient-specific formulations. Similarly, bioinks often lack the optimal combination of viscosity, mechanical strength, and biological functionality, creating a trade-off between printability and cell viability. Advancing the design of hybrid bioinks and stimuli-responsive (“4D”) materials represents a key research direction [36].

Another challenge lies in achieving vascularization and scalability of printed constructs. Current techniques are often limited by the diffusion barrier of oxygen and nutrients, restricting the size and functionality of tissues. Research is increasingly focused on sacrificial bioinks, computational modeling of oxygen distribution, and hierarchical vascular design to enable clinically relevant constructs. Parallel to this, scalability requires innovations in automated, high-throughput bioprinting systems that can ensure reproducibility and cost-effectiveness without compromising biological integrity [50].

Quality control and standardization represent additional hurdles. Minor variations in printing parameters can lead to inconsistent drug release profiles or cell responses, complicating regulatory approval. Establishing reproducible protocols for rheological testing, cytocompatibility, and long-term performance is an urgent research priority. Collaborative efforts, such as ASTM International’s guidelines for bioinks, mark a positive step, but further work is needed to harmonize biological and mechanical testing standards across laboratories [50].

The long-term integration of 3D and 4D bioprinted scaffolds in skin, bone, and other tissues also faces fundamental barriers. Material-related limitations, such as the restricted availability of multifunctional biomaterials that are simultaneously biocompatible, biodegradable, and stimuli-responsive, hinder sustained in vivo performance. Biological challenges, including inadequate vascularization, immune rejection, and unpredictable cell–material interactions, further compromise integration. Technical issues such as the trade-off between printing resolution and cell viability, the lack of predictive computational models, and difficulties in large-scale reproducibility also pose obstacles. Finally, regulatory hurdles, high fabrication costs, and the absence of standardized long-term evaluation protocols delay clinical translation [124]. Addressing these challenges will be essential to achieve durable scaffold integration and successful tissue regeneration.

Artificial intelligence (AI) is likely to become an important driver of future advances in bioprinting. It can help optimize the scaffold geometry, material choice, and printing parameters, while also enabling personalized designs from patient-specific data. Coupled with automation and real-time monitoring, AI has the potential to improve the reproducibility and scale-up production of complex constructs. The main challenges ahead include the need for reliable datasets, unpredictable biological responses, and limited transparency of AI models. Addressing these issues will be key to safe clinical translation [127,128,129].

In summary, future research should focus on developing advanced biomaterials, scalable bioprinting platforms, and robust standardization frameworks, while harnessing computational tools such as AI and machine learning. These efforts will be essential to overcome the existing barriers and translate 3D printing technologies from experimental research into mainstream pharmaceutical and clinical applications.

## 10. Conclusions

Overall, 3D printing is transforming the development of patient-specific medical devices and tissue constructs, but its successful clinical use depends on addressing several key challenges. A clear understanding of the available printing techniques and their limitations is essential for selecting suitable methods for specific applications. Biomaterial selection plays a crucial role, requiring careful evaluation of the consistency, compatibility, degradation, and surface interactions. Regulatory compliance, including sterility and mechanical standards, is necessary to ensure product safety and performance. The technology still faces barriers such as limited excipient availability, hardware and software limitations, high initial costs, and unresolved intellectual property concerns. Advancements in materials, printer design, and standardization are gradually improving the feasibility of 3D printing in healthcare. Focusing on process reliability, appropriate material use, and regulatory clarity will support broader adoption and enable progress in personalized medical treatments.

## Figures and Tables

**Table 2:** Comparative properties of natural, synthetic, and hybrid (composite) bioinks in scaffold fabrication [50].

Aspect	Natural Bioinks	Synthetic Bioinks	Hybrid (Composite) Bioinks
Strengths	High biocompatibility with minimal immune rejection. Provide inherent bioactive signals (e.g., RGD motifs) that support cell adhesion, proliferation, and differentiation. Naturally biodegradable, breaking down into non-toxic products. Closely mimic the extracellular matrix (ECM), creating a physiologically relevant microenvironment.	Properties can be tailored with precision, including the degradation rate, elasticity, and mechanical strength. Offer excellent reproducibility and batch-to-batch consistency. Provide superior mechanical stability and durability. Highly processable using advanced fabrication methods (3D printing, electrospinning, molding).	Combine the bioactivity of natural polymers with the tunability and robustness of synthetics. Provide enhanced printability, shape fidelity, and structural integrity. Support long-term mechanical stability while maintaining biological cues for tissue-specific functions.
Limitations	Variable composition depending on the biological source. Limited mechanical strength, restricting use in load-bearing tissues. Potential immunogenicity, particularly for animal-derived materials. Degradation may be difficult to precisely control. Processing into complex shapes can be challenging.	Lack intrinsic bioactivity, requiring surface modification or blending with bioactive molecules. Some may trigger immune reactions or release harmful by-products. Hydrophobicity can hinder nutrient diffusion and cell adhesion. Degradation can release acidic by-products, leading to inflammation.	Fabrication often requires complex processing and crosslinking. Risk of phase separation or inconsistent mixing between natural and synthetic components. Production can be costly. Optimization is needed to maintain a balance between mechanical and biological properties.

## Data Availability

No new data were created in this study. Data sharing is not applicable to this article.
